# Populations and assemblages living on the edge: dung beetles responses to forests-pasture ecotones

**DOI:** 10.7717/peerj.6148

**Published:** 2018-12-13

**Authors:** Ana Paola Martínez-Falcón, Gustavo A. Zurita, Ilse J. Ortega-Martínez, Claudia E. Moreno

**Affiliations:** 1 Centro de Investigaciones Biológicas-Instituto de Ciencias Básicas e Ingenierías, Universidad Autónoma del Estado de Hidalgo, Mineral de la Reforma, Hidalgo, Mexico; 2 Instituto de Biología Subtropical-Facultad de Ciencias Forestales, Universidad Nacional de Misiones-CONICET, Puerto Iguazú, Misiones, Argentina

**Keywords:** Dung beetles, Ecotone, Pasturelands, Community ecology, Population ecology, Juniperus, Pine-oak forests, The Mexican Transition Zone, Functional diversity, Hill numbers

## Abstract

Edge effects alter insect biodiversity in several ways. However, we still have a limited understanding on simultaneous responses of ecological populations and assemblages to ecotones, especially in human modified landscapes. We analyze edge effects on dung beetle populations and assemblages between livestock pastures and native temperate forests (*Juniperus* and pine-oak forests (POFs)) to describe how species abundances and assemblage parameters respond to edge effects through gradients in forest-pasture ecotones. In *Juniperus* forest 13 species avoided the ecotones: six species showed greater abundance in forest interior and seven in pasturelands, while the other two species had a neutral response to the edge. In a different way, in POF we found five species avoiding the edge (four with greater abundance in pastures and only one in forest), two species had a neutral response, and two showed a unimodal pattern of abundance near to the edge. At the assemblage level edge effects are masked, as species richness, diversity, functional richness, functional evenness, and compositional incidence dissimilarity did not vary along forest-pasture ecotones. However, total abundance and functional divergence showed higher values in pastures in one of the two sampling localities. Also, assemblage similarity based on species’ abundance showed a peak near to the edge in POF. We propose that conservation efforts in human-managed landscapes should focus on mitigating current and delayed edge effects. Ecotone management will be crucial in livestock dominated landscapes to conserve regional biodiversity and the environmental services carried out by dung beetles.

## Introduction

Despite an extensive body of research on edge effects as key processes influencing individuals, populations, ecological interactions, communities and ecosystem processes (Murcia, 1995; Ries et al., 2004; Porensky, 2011; Ewers, Bartlam & Didham, 2013; Ziter, Bennett & Gonzalez, 2014), relatively few studies have focused on simultaneous responses to ecotones using a unified conceptual and analytical framework (Ries et al., 2004; Ries & Sisk, 2004; Ewers & Didham, 2006; Porensky, 2011). Several ecological mechanisms involved in edge effects, including differential availability of resources, dispersal limitations, and ecological filtering, result in a large variety of responses among populations and communities (Magura & Lövei, 2017). Thus, understanding responses to edge effects is a main challenge for ecologists, especially in human modified landscapes where several studies have demonstrated striking edge-related shifts in plant and animal populations and communities (Murcia, 1995; Laurance et al., 2002; Santos et al., 2010).

In a seminal paper, Ewers & Didham (2006) developed an analytical approach that quantifies both the magnitude and extent of edge effects considering distance as a continuous variable, in both sides of the interior-exterior gradient (Fonseca & Joner, 2007). A series of recent studies using this continuous approach showed that species might have an unimodal response near to or at the edge, edge avoidance (sigmoid or linear response) or edge insensitivity (neutral response), and this framework can be applied to both complete (covering the entire range of distances) and incomplete biological responses to edge effects. This approach has been recently used to assess edge effects in fragmented landscapes where native habitats such as forests have been converted into pastures and agricultural lands uses, to assess population responses in ecotones between forest fragments and novel agroecosystems (Campbell et al., 2011; Ewers, Bartlam & Didham, 2013; Peyras et al., 2013; Barnes et al., 2014; Villada-Bedoya et al., 2017).

Pasturelands for livestock raising are among the most degrading land uses because they usually convert highly biodiverse forests into pastures dominated by few grass species (Ospina et al., 2012), causes erosion and compactness of the soils (Herrero et al., 2013), and livestock is an important emitter of greenhouse gases (about 20% of total emissions globally, O’Mara, 2011). In cattle pastures dung beetles (Coleoptera: Scarabaeinae) play an important role burying large amounts of livestock dung (Hanski & Cambefort, 1991), and this ecosystem service provides huge economic benefits, mainly by maintaining clean areas (López-Collado et al., 2017). Dung beetles evolved feeding mainly on mammalian dung and use the fibrous material to brood their larvae (Halffter & Edmonds, 1982). By doing this, dung beetles also play other important functions in ecosystems including seed dispersal, soil aeration, nutrient cycling, soil mineralization, carbon sequestration, and mitigation of greenhouse emissions (Yamada et al., 2007; Nichols et al., 2008; Penttilä et al., 2013; López-Collado et al., 2017). Moreover, dung beetles can respond rapidly to land use changes and landscape alterations derived from human activities (Larsen, Lopera & Forsyth, 2008; Beiroz et al., 2017; Lascaleia et al., 2018). Altogether, in lowland tropical ecosystems there are well-documented patterns on the decrease of dung beetle diversity due to the transformation of forest habitats into pastures (Nichols et al., 2008).

The Mexican Transition Zone (MTZ; Halffter, 1964; Morrone, 2015; Halffter & Morrone, 2017) in central Mexico has an intricate geological history and is one of the most biologically diverse regions of the world, where Nearctic and Neotropical biotas with different evolutionary histories got in contact. In this region, dung beetles from different historical origins currently coexist, including Neotropical, Mesoamerican, Paleoamerican, and Plateau origins (Halffter, 1964), and recently there are also African exotic species (Montes-De-Oca & Halffter, 1998). This complex biogeographic history causes particular responses of dung beetle fauna to land use conversion in this region. Contrary to their observed response in tropical ecosystems, in montane forests of the MTZ livestock may promote dung beetle diversity and abundance (Barragán et al., 2014; Moctezuma, Halffter & Escobar, 2016; Ortega-Martínez, Moreno & Escobar, 2016). For example, in pine-oak forests (POFs) of the Sierra Madre Oriental and in xeric scrublands of the Mexican Plateau, grazed areas have promoted the presence and abundance of dung beetles (Barragán et al., 2014). Also, in spite of having similar species richness, the abundance of beetles and amount of dung removed by beetle assemblages on pastures were higher than in native *Juniperus* forest (JF) remnants (Ortega-Martínez, Moreno & Escobar, 2016), and in a temperate mountain with mixed forest, disturbed habitats may be twice as diverse as conserved habitats (Moctezuma, Halffter & Escobar, 2016).

The variety of dung beetle responses to land use conversion in this region offers the possibility of detecting several edge effects, as a consequence of different land-use impacts on biodiversity patterns. Thus, the aim of this study was to analyze edge effects on dung beetle populations and assemblages between grazed areas (sheep and cattle pasturelands) and two native temperate forests types of the MTZ (*Juniperus* and POFs). We address the following objectives: (a) to describe populations’ abundance responses to edge effects (edge avoidance, neutral, or unimodal response) through gradients in forest-pasture ecotones, and (b) to assess assemblage’s responses (species richness, abundance, species diversity, functional diversity, and similarity in species composition) through the same gradients. We predict that: (1) most of the species will tend to avoid ecotones and increase their abundance in their preferred habitat (either pasture or forest), (2) species richness, total abundance, species diversity, and functional diversity at the assemblage level will be higher in pasturelands due to the biogeographical histories of species in this region (in accordance with the findings of Barragán et al., 2014; Moctezuma, Halffter & Escobar, 2016; Ortega-Martínez, Moreno & Escobar, 2016), and (3) due to the coexistence of forest and pasture adapted species, dissimilarity in species composition will be higher in ecotones (Villada-Bedoya et al., 2017), showing an unimodal response.

## Materials and Methods

### Study area

**Figure 1:**
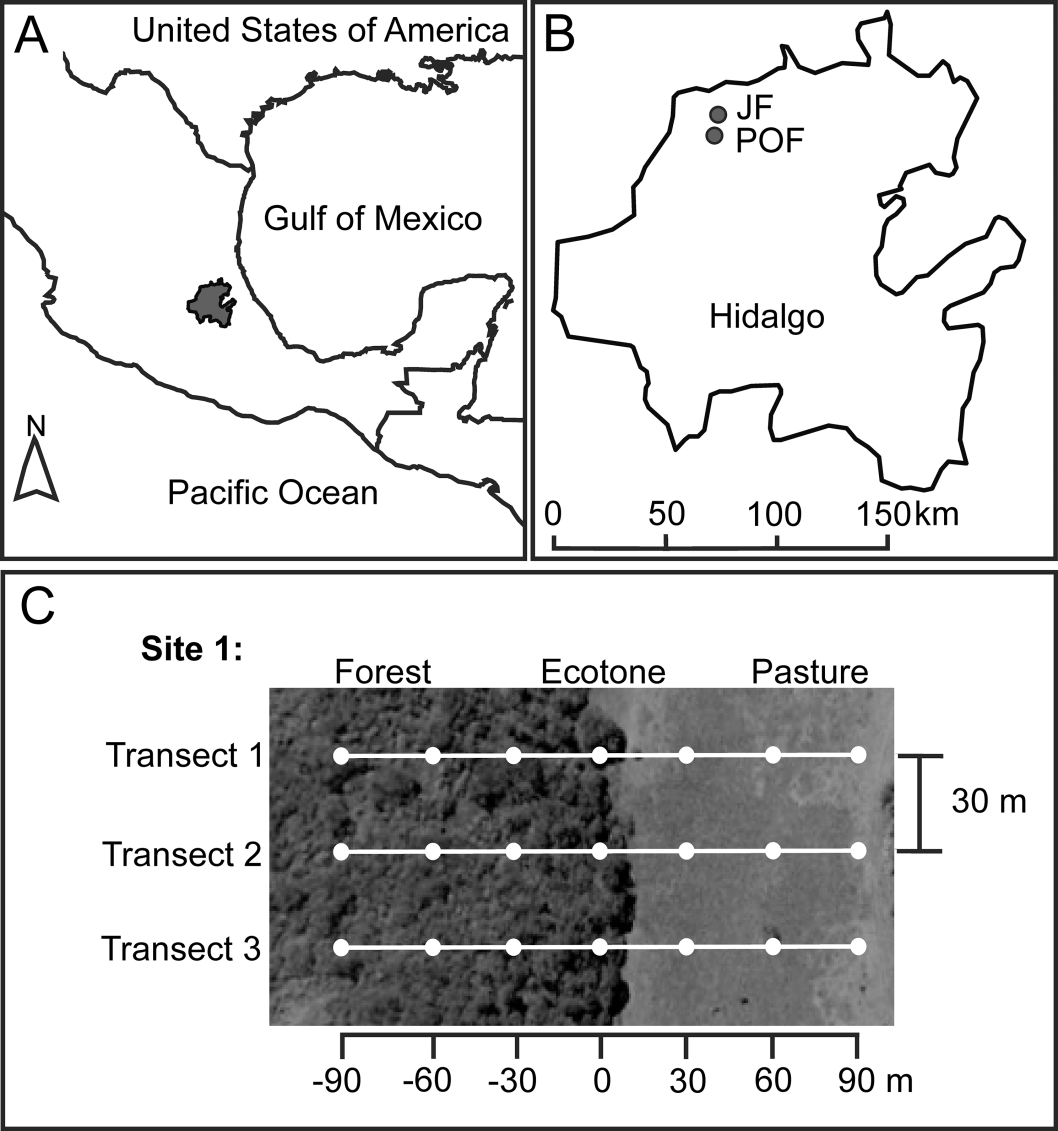
Location of Hidalgo state in the Mexican Transition Zone, Mexico (A), and two sampling localities (B) with *Juniperus* sp. forest (JF) and pine-oak forest (POF). For each forest type we located two sampling sites, and here we exemplified the sampling design for one of these sites (C). White dots represent pitfall traps at seven distances along three transects from the forest interior (−90 m) toward the pasture (90 m), with the ecotone set as zero.

The study was carried out in two localities with different temperate forest types: JF and POFs, in the state of Hidalgo, Mexico, in the MTZ (Fig. 1). In both localities, forest fragments are imbedded in pasture matrices where sheep, horses, and cattle graze. Mean annual precipitation in this region is ca. 2,000 mm, and the rainy season occurs from June to October, with a temperate climate (Pavón & Meza, 2009).

The JF locality (21°1′1.17″N, 99°12′2.35″W) is dominated by *Juniperus flaccida* Schltdl., *J. deppeana* Steud., and *Cupressus* sp. Mean elevation is 1,376 m a.s.l. and mean temperature during dung beetle sampling (see below) was 23.11 °C (31.52 °C maximum; 17.90 °C minimum) in native vegetation, and 24.74 °C (34.01 °C maximum; 17.52 °C minimum) at pastures. The POF locality (20°56′36.65″N, 99°12′29.23″W) is dominated by *Pinus teocote* Schiede ex Schltdl. & Cham., *Pinus montezumae* Lamb., *Pinus cembroides* Zucc., *Quercus mexicana* Bonpl. and *Q. crassifolia*, Bonpl. Mean elevation is 1,850 m a.s.l. and average temperature during the dung beetle sampling was 17.97 °C (27.12 °C maximum; 14.85 °C minimum) in native vegetation and 18.01 °C (25.17 °C maximum; 14.47 °C minimum) at pastures.

### Sampling design

We selected two sampling sites at each locality. The two sites in JF were separated 480 m, and the two sites in POF were 530 m apart. Each site has a forest remnant (mean size of fragments: eight ha) surrounded by pastures. At each site, we located three linear transects, starting 90 m inside the native forest and ending 90 m into the pastureland, and each transect had seven baited pitfall traps (see below) separated 30 m (Fig. 1). Distances of traps from the ecotone toward the forest interior are indicated with negative values (−30, −60, −90 m), distances of traps from the ecotone toward the pasture with positive values (30, 60, 90 m), and the ecotone trap is considered as zero distance, set under the tree line (Fig. 1). A recent study on the dung beetle assemblage and species with the highest numbers of recaptured individuals in the Atlantic forest, Brazil, suggest a minimum distance of 100 m between pairs of traps to minimize interference between baited pitfall traps (Da-Silva & Medina-Hernández, 2015). However, a mark–recapture experiment in Venezuela found that 95% of the recaptured beetles were attracted from within 26.20 m of the trap (Larsen & Forsyth, 2005). Therefore, given the environmental contrast in the adjacent habitats studies, we assume that the beetles at each trap are reliable representatives of the distance to the ecotone.

Pitfall traps were baited with a mixture (3:1) of sheep and horse dung (ca. 250 g), as this method has been successfully used in the region for sampling dung beetles, given that the native mammal fauna was best represented by herbivorous like white-tailed deer, but currently wild mammals are scarce and the main source of dung is livestock (Barragán et al., 2014). Each pitfall trap was a plastic container (18.5 cm diameter, one L capacity) buried in the soil, containing ethylene-glycol to break the surface tension and preserve the beetles. Fresh dung was supported on a wire grid at the top of the container. To increase sampling completeness, we carried out this procedure twice (July and August 2008) and for each trap we summed the captures to obtain the cumulative number of beetles. This is the middle of the rainy season, when insects are most active and thus samplings in these months can maximize capture success of dung beetles in the region (Ortega-Martínez, Moreno & Escobar, 2016). Traps were active for 48 h each time.

Therefore, our analyses are based on the beetles that are attracted to the traps for food at close distances to the ecotone, and we assume that their identity and abundance at each sampling distance to the edge represents their foraging patterns near the ecotone. This sampling method is usual in dung beetle studies, although beetles may fly toward the trap but prefer different conditions for nesting in the surroundings. So, with this sampling method we do not aim to assess reproductive populations, but the flying beetles that can arrive for food near to the forest edge.

Dung beetles were preserved in alcohol, and some specimens were pinned and identified to species using taxonomic keys (Delgado, Pérez & Blackaller, 2000; Morón, 2003; Vaz-De-Mello et al., 2011) and comparing with voucher specimens from a local reference collection and with the help of Fernando Escobar (Instituto de Ecología, A.C., Xalapa, México).

### Data analysis

#### Population abundance

To assess population responses to edge effects along the forest-pasture gradient with a sufficient number of individuals, for each site we joined the number of individuals captured in the traps of the same distance (corresponding to the three transects) as a single sampling unit (SU). Differences among transects are minimal (see Results “Population responses to the edge”); however, these differences cannot be statistically verified because of the low number of transects, which limits the power of any analysis. Thus, for each site we retained seven SU (at −90, −60, −30, 0, 30, 60, and 90 m). We calculated the relative abundance of each dung beetle species at each SU as the number of individuals of the species divided by the total number of individuals.

#### Assemblage parameters

First, to assess the completeness of dung beetle assemblage inventories for the 28 SUs (7 SUs × 4 sites) we calculated the sample coverage (Chao & Jost, 2012). Then, we calculated the following metrics: (a) species richness, (b) species diversity, (c) functional divergence (FDiv), (d) functional richness (FRic), (e) functional evenness (FEve), and (f) compositional dissimilarity. Species richness (*S*) was measured as the number of species observed at each site. Species diversity (*D*) was measured using the exponential of Shannon’s entropy, which is the true diversity of order *q* = 1 (sensu Jost, 2006).

We used three indices that measure complementary aspects of functional diversity (Villéger, Mason & Mouillot, 2008; Mouchet et al., 2010): FDiv describes how species’ abundance is spread within the functional trait space occupied by all species; FRic is the volume of functional space filled by the assemblage; and FEve is the regularity with which the functional space is filled by species, weighted by their abundance. These metrics were calculated in the FDiverstiy software (Casanoves et al., 2010) using four functional traits: food relocation behavior (species can be rollers, tunnelers or dwellers), activity period (diurnal or nocturnal), diet (strictly coprophagous or copro-necrophagous) and biomass (dry weight) (Table S1). Roller species shape the food source into a ball and roll it on the ground to another location for burial, tunneler species build their nests and bury portions of food in tunnels beneath the resource, while dwellers breed inside the dung itself (Hanski & Cambefort, 1991). For diet, coprophagous are those species that feed on dung, while copro-necrophagous are attracted to dung but also carrion. Functional traits of the species were obtained from specialized literature (Halffter & Edmonds, 1982; Hanski & Cambefort, 1991; Pineda et al., 2005; Navarrete & Halffter, 2008; Barragán et al., 2011). We calculated mean biomass of each beetle species as the dry weight of 50 randomly selected individuals per species. For species with sexual dimorphism we weighted 25 males and 25 females. To obtain dry weight we put individuals in an oven at 70 °C for 48 h. After that, we weighed beetles in a digital scale (Scientech ZSA 80, precision ± 0.001 g).

The response of compositional dissimilarity to the edge was measured with a dissimilarity index based on presence–absence (1-Jaccard similarity) and an abundance-based index (1-Morisita similarity). To get their values, we compared species composition of each SU with their two adjacent SU in the gradient and calculated average dissimilarity, except for the extreme SU (−90 and 90 m) where we took one dissimilarity value with the unique adjacent SU (−60 or 60 m, respectively). We expect the response of dissimilarity to edge effects to be clearer with the abundance-based index than with the presence–absence index.

#### Assessment of edge effects

The response of populations and assemblage parameters to the edge was analyzed using the procedure of Ewers & Didham (2006) modified by Zurita et al. (2012). Our population analyses were restricted to those species with more than 30 individuals captured on each forest type. We used five theoretical models that represent the three expected edge responses: (1) neutral (mean equation), (2) edge avoidance (linear, power or sigmoid), and (3) unimodal response. The mean model describes species that use both habitats equally and therefore exhibit no response to edges (generalist species). Regarding edge avoidance, the linear model would best describe circumstances where the response of species to edges extends beyond the sampled range on both sides of the ecotone; the power function may be useful for describing the incomplete coverage of the edge response of species in which an asymptote is reached on one side of the ecotone; and the sigmoid model describes circumstances in which species respond to edges either gradually or abruptly, and there is thus a discrete change in habitat suitability. Finally, the unimodal response fits edge preference, where species have the highest abundance at the middle of the gradient (Zurita et al., 2012). In this framework, the neutral response corresponds to “reflecting edges,” and edge preference to “absorbing edges” *sensu* Olson & Andow (2008). Our sampled distances (−90 to 90 m) could encompass the full extent of these edge effects (sigmoid and unimodal) showing a complete response, or edge effect could extend larger distances in both habitats (linear) or in one habitat (power) indicating an incomplete response. Basically, we used non-linear regression analyses to test for these models, using the relative abundance of species (the number of individuals of each species divided by the total number of individuals of each site) or the assemblage parameters as the dependent variables, and the distance to the nearest edge type as the independent variable. The full description and statistical procedures can be found in the proposal of Ewers & Didham (2006), Zurita et al. (2012) and Peyras et al. (2013).

To compare the best fit of each dependent variable (species relative abundance and assemblage parameters) to the five proposed models (neutral, linear, power, sigmoid, unimodal) we first calculated the Akaike’s information criteria for each model, with a correction for small sample sizes (AICc). Then, we selected the two most probable models (lower AICc) and calculated the Akaike weight between both models (−0.5 * ΔAIC). The Akaike weight represents the relative likelihood of a model (probability of being correct). Finally, we selected the model with more than 90% of probability of being correct. In cases were both models had more than 90% probability of being correct we keep both models as the most probable responses to the edge. In cases when a complete (sigmoid or unimodal) and an incomplete model (linear or power) showed similar fit the final response was considered undetermined.

Summarizing, the response of dung beetles could be (1) unimodal distribution of abundance with a peak near to the edge, (2) edge avoidance (linear, power or sigmoid) or (3) neutral response (mean model). The extent of the response can be incomplete if edge effects extend beyond the 90 m sampled distances (linear or power) or complete (sigmoid and unimodal). In cases when a complete and incomplete model showed similar fit, the response is considered undetermined. Finally, to compare the proportional number of species fitting to each model between localities, we performed a *G*-test.

## Results

We collected 41,636 individuals belonging to 22 species. From this total, we found 33,431 individuals from 20 species in JF, and 8,205 individuals from 16 species in POF. *Canthon humectus hidalgoensis* Bates, 1887 (*n* = 9,729), *Onthophagus incensus* Say, 1835 (*n* = 8,534) and *O. knulli* Howden & Cartwright, 1963 (*n* = 6,350) were the most abundant species in JF, while the most abundant species in POF were *O. mexicanus* Bates, 1887 (*n* = 4,356), *O. incensus* (*n* = 2,284), and *Phanaeus adonis* Harold, 1863 (*n* = 506) (Table S1). Overall, dung beetles were more abundant in pasturelands (16,455 individuals in the JF locality, 6,285 individuals in the POF locality) than in forests (12,151 individuals in JF, 1,179 individuals in POF). The majority of species are tunnelers (26,201 individuals from 13 species), 15,399 individuals from eight species are rollers, and only one species (*Eurysternus magnus* Laporte De Castelnau, 1840, with 36 individuals) is considered dweller. Sample coverage was close to 100% in all SUs (Table S2).

### Population responses to the edge

**Figure 2:**
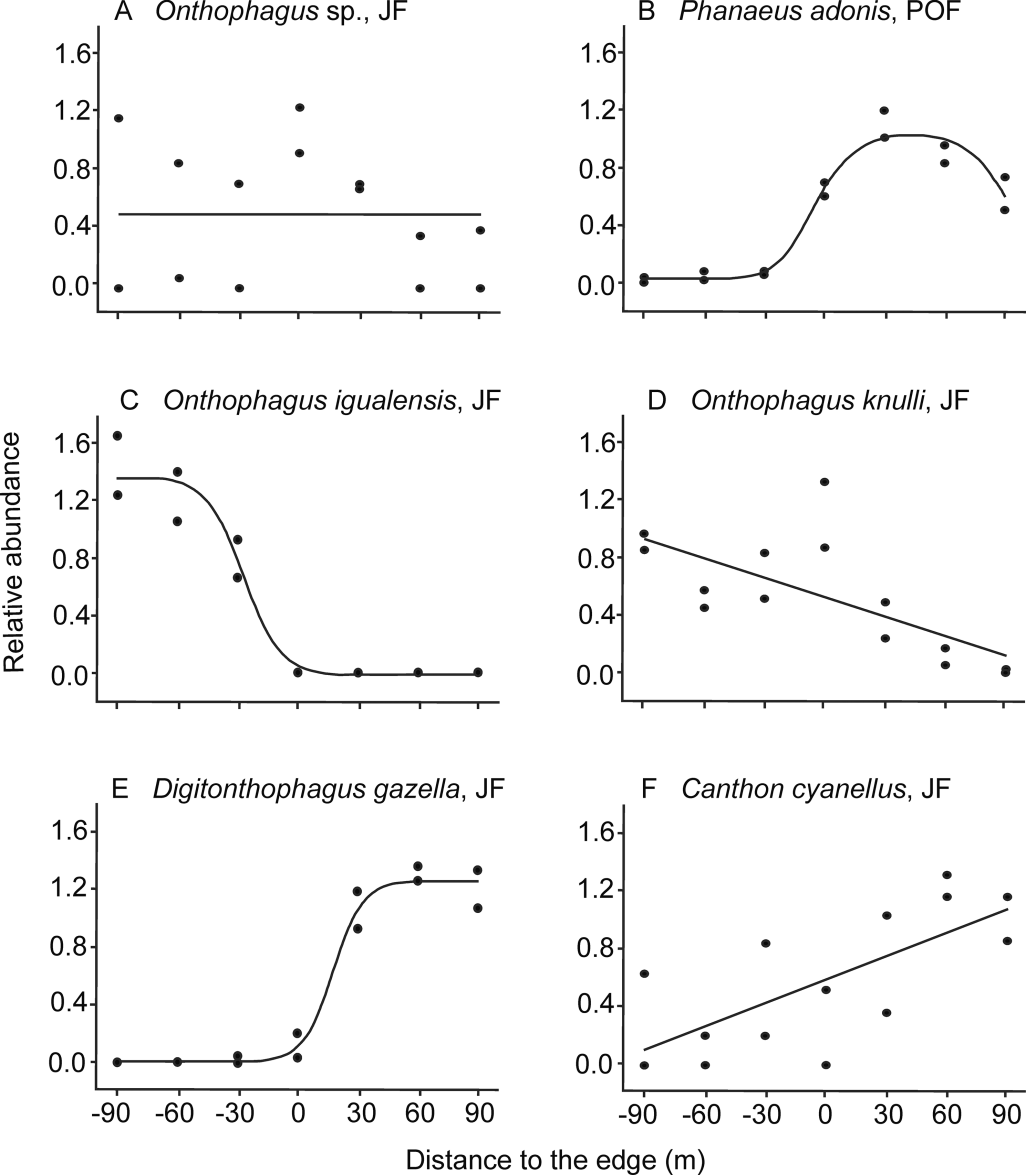
Examples of dung beetle species’ responses to the forest-pasture ecotones, based on their relative abundance. Negative values indicate distances to the edge inside forest, while positive values indicate distances inside pasture. (A) Neutral response (mean model) observed for *Onthophagus* sp. in *Juniperus* forest (JF); (B) unimodal response of *Phanaeus adonis* in pine-oak forest (POF); (C) edge avoidance of *Onthophagus igualensis* (complete response) and (D) *Onthophagus knulli* (incomplete response) showing preference for the forest interior; (E) edge avoidance of *Digitonthophagus gazella* (complete response) and (F) *Canthon cyanellus* (incomplete response) with higher abundance in pastures.

We analyzed the response to the edge of 18 species that had more than 30 individuals captured (24 cases: 15 species in JF and nine species in POF, Table 1; details of model fitting are in Table S3, the variation of abundance among transects of each site is in Table S4). From these, three species had a neutral response to the edge, that is, without a clear preference for any habitat condition: *Copris incertus* Say, 1835 in both forests, *E. magnus* Laporte De Castelnau, 1840 in POF, and *Onthophagus* sp. in JF (Fig. 2A). *Canthon humectus* and *Phanaeus adonis* responded otherwise, with a unimodal distribution of abundance in POF (Fig. 2B), although both species were more abundant in the pastures of JF.

**Table 1:** Response of dung beetle populations and assemblages to ecotones between pastures and *Juniperus* (JF) or pine-oak (POF) forest in the Mexican Transition Zone. Responses to the edge are neutral (mean model), unimodal or avoidance.

	Forest type	Mean	Linear		Power		Sigmoid		Unimodal		Akaike weights (%)		Response	Highest abundance
		AICc	AICc	*R*^2^	AICc	*R*^2^	AICc	*R*^2^	AICc	*R*^2^	Second	Best		
**Population responses**														
*Copris incertus* Say, 1835	JF	**−16.3**	−16.2	0.20	−15.5	0.16	−11.6	0.42	−5.1	0.42	47.7	52.3	Neutral	
*Copris incertus* Say, 1835	POF	**−25.2**	−22.0	0.01	−22.0	0.01	NC	–	NC	–	17.0	83.0	Neutral	
*Eurysternus magnus* Laporte De Castelnau, 1840	POF	**−18.7**	−15.7	0.02	−16.0	0.04	−14.0	0.42	−11.5	0.57*	20.4	79.6	Neutral	
*Onthophagus* sp.	JF	**−14.9**	−12.3	0.05	−12.2	0.04	−5.9	0.21	−1.4	0.22	21.7	78.3	Neutral	
*Canthon* (*Canthon*) *humectus hidalgoensis* Bates, 1887	POF	−9.5	−13.1	0.38	−10.8	0.39	−11.8	0.65*	**−19.3**	0.87*	4.4	95.6	Unimodal	
*Phanaeus* (*Phanaeus*) *adonis* Harold, 1863	POF	−16.5	−26.2	0.61*	−22.1	0.47*	−33.2	0.87*	**−41.6**	0.96*	1.5	98.5	Unimodal	
*Onthophagus igualensis* Bates, 1887	JF	−8.8	−27.4	0.79*	−31.2	0.84*	**−40.6**	0.96*	−35.0	0.96*	5.8	94.2	Avoidance, C	Forest
*Onthophagus incensus* Say, 1835	JF	−37.3	**−39.5**	0.32*	**−39.0**	0.30	−35.1	0.52*	−28.6	0.52*	44.5	55.5	Avoidance, I	Forest
*Sysiphus mexicanus* Harold, 1863	JF	−18.2	**−49.2**	0.91*	−39.6	0.83*	−44.5	0.94*	−39.1	0.94*	8.9	91.1	Avoidance, I	Forest
*Deltochilum scabriusculum* Bates, 1887	JF	−13.2	**−22.2**	0.58*	−18.8	0.46*	**−19.5**	0.73*	−13.0	0.73*	21.1	78.9	Avoidance, U	Forest
*Onthophagus knulli* Howden & Cartwright, 1963	JF	−24.9	**−31.0**	0.49*	−29.2	0.42*	**−29.9**	0.71*	−23.9	0.71*	36.5	63.5	Avoidance, U	Forest
*Onthophagus* sp.	POF	−29.1	**−35.7**	0.5*	**−35.0**	0.48*	−29.8	0.61*	−24.2	0.63*	41.5	58.5	Avoidance, I	Forest
*Canthon* (*Boreocanthon*) *puncticollis* LeConte, 1866	JF	−8.5	−21.8	0.69*	−20.8	0.67*	**−26.6**	0.88*	−20.1	0.88*	8.5	91.5	Avoidance, C	Pasture
*Canthon* (*Canthon*) *humectus hidalgoensis* Bates, 1887	JF	−20.3	−33.8	0.70*	−33.4	0.69*	**−48.3**	0.94*	−42.2	0.94*	4.6	95.4	Avoidance, C	Pasture
*Canthon* (*Canthon*) *imitator* Brown, 1946	JF	−16.4	32.0	0.74*	−31.6	0.72*	**−52.1**	0.97*	−45.9	0.96*	3.3	96.7	Avoidance, C	Pasture
*Digitonthophagus gazella* Fabricius, 1787	JF	−10.3	−28.9	0.79*	−25.8	0.74*	**−51.3**	0.98*	−44.8	0.98*	3.9	96.1	Avoidance, C	Pasture
*Canthon cyanellus cyanellus* LeConte, 1859	JF	−16.9	**−23.4**	0.50*	**−24.0**	0.52*	−18.5	0.63*	−12.0	0.63*	43.5	56.5	Avoidance, I	Pasture
*Dichotomius colonicus* Say, 1835	JF	−23.3	**−25.8**	0.34*	**−25.1**	0.31*	−20.1	0.48	−13.6	0.48	41.4	58.6	Avoidance, I	Pasture
*Glaphyrocanthon* sp.	JF	−17.6	**−31.2**	0.70*	**−32.0**	0.70*	−25.5	0.76*	−20.0	0.78*	40.4	59.6	Avoidance, I	Pasture
*Phanaeus* (*Phanaeus*) *adonis* Harold, 1863	JF	−25.2	**−37.6**	0.67*	**−35.2**	0.61*	−33.3	0.77*	−27.0	0.77*	23.1	76.9	Avoidance, I	Pasture
*Onthophagus mexicanus* Bates, 1887	POF	−12.4	−26.3	0.71*	−23.2	0.63*	**−27.6**	0.86*	−21.2	0.86*	9.9	90.1	Avoidance, C	Pasture
*Onthophagus incensus* Say, 1835	POF	−41.0	**−56.8**	0.74*	**−54.1**	0.69*	−53.0	0.83*	−46.5	0.82*	20.3	79.7	Avoidance, I	Pasture
*Onthophagus gibsoni* Howden & Génier 2004	POF	−15.3	**−30.7**	0.73*	−25.6	0.62*	**−29.1**	0.85*	−26.4	0.88*	30.6	69.4	Avoidance, U	Pasture
*Onthophagus knulli* Howden & Cartwright, 1963	POF	−21.6	**−45.8**	0.86*	−39.0	0.77*	**−45.4**	0.92*	−39.3	0.93*	44.9	55.1	Avoidance, U	Pasture
**Community responses**														
Species richness	JF	**19.5**	19.4	0.21	19.5	0.21	26.6	0.31	35.1	0.21	48.1	51.9	Neutral	
Species richness	POF	**17.7**	19.2	0.12	19.3	0.11	25.6	0.28	32.6	0.25	31.4	68.7	Neutral	
Abundance	JF	175.5	**163.6**	0.66*	166.2	0.59*	**162.0**	0.84*	179.2	0.66*	31.4	68.6	Avoidance, I	Pasture
Abundance	POF	**195.0**	197.4	0.06	197.4	0.06	205.3	0.14	NC	–	23.6	76.5	Neutral	
Diversity	JF	**1.9**	4.9	0.02	4.9	0.01	12.31	0.14	16.3	0.27	18.2	81.8	Neutral	
Diversity	POF	**−9.4**	−6.9	0.06	−6.9	0.06	1.5	0.10	NC	–	23.0	77.0	Neutral	
FRic	JF	**41.9**	42.6	0.17	42.8	0.16	51.4	0.18	NC	–	41.4	58.5	Neutral	
FRic	POF	**52.3**	53.1	0.17	53.5	0.14	56.2	0.45	59.6	0.56*	40.6	59.4	Neutral	
FEve	JF	**−65.5**	−65.7	0.21	−65.7	0.21	−56.6	0.22	−50.1	0.2	49.9	50.0	Neutral	
FEve	POF	**−61.0**	−57.7	0.00	−57.7	0.00	NC	–	NC	–	16.2	83.8	Neutral	
FDiv	JF	**−63.4**	−60.1	0.00	−60.1	0.00	NC	–	NC	–	16.1	83.8	Neutral	
FDiv	POF	−69.4	**−73.2**	0.40*	**−73.3**	0.40*	−65.6	0.46	−59.1	0.46	49.6	50.4	Avoidance, I	Pasture
Jaccard dissimilarity	JF	**−55.9**	−57.9	0.00	−58.0	0.00	−56.0	0.09	−44.5	0.26	NC	NC	Neutral	
Jaccard dissimilarity	POF	**−68.2**	−64.9	0.00	−60.9	0.00	−57.2	0.09	−53.5	0.26	NC	NC	Neutral	
Morisita dissimilarity	JF	**−69.3**	−66.7	0.00	−61.7	0.00	−60.4	0.21	−56.8	0.36	NC	NC	Neutral	
Morisita dissimilarity	POF	**−54.4**	−51.1	0.00	−47.0	0.00	−44.2	0.16	**−56.3**	0.97*	99.9	99.9	Unimodal	

**Notes:**

For avoidance we indicate if the response was complete (C: sigmoid model), incomplete (I: linear or power), or undetermined (U), when a complete and an incomplete model showed similar fit, as well as the habitat with highest number of individuals. All possible responses to the edge were compared using the AICc. Additionally, the probability of being correct (Akaike weight) was calculated between the two most probable models (best and second).

Bold numbers correspond to first, or first and second best models.

NC, Not calculated.

* Significant models (α < 0.05).

As predicted, most of the species avoided ecotones: 87% of the species in JF and 56% in POF (Table 2). On one hand, six populations were more abundant toward the forest interior, five of them in the JF (Table 1): *O. igualensis* Bates, 1887 (with a complete response within our gradient, Fig. 2C), *O. incensus*, *Sysiphus mexicanus* Harold, 1863 (both with an incomplete response), *Deltochilum scabriusculum* Bates, 1887 and *O. knulli* (with undetermined avoidance response, Fig. 2D). In the POF only *Onthophagus* sp. showed an incomplete edge avoidance response and higher abundance within the forest interior.

**Table 2:** Percentage and number of dung beetle species (in parenthesis) with different responses to *Juniperus* (JF) and pine-oak (POF) forest-pasture ecotones in two localities of the Mexican Transition Zone, according to the type of response to the edge.

Type of Response	Total	JF	POF
Edge avoidance	75% (18)	87% (13)	56% (5)
Neutral response	17% (4)	13% (2)	22% (2)
Unimodal response	8% (2)	0% (0)	22% (2)
**Extent of the response**			
Complete	40% (8)	38% (5)	43% (3)
Incomplete	40% (8)	46% (6)	29% (2)
Undetermined	20% (4)	15% (2)	29% (2)

**Note:**

Edge avoidance: sigmoid, power or linear models; Neutral: mean model; Complete: edge effects extend less than 90 m; Incomplete: edge effects extend beyond 90 m.

On the other hand, in 12 cases populations avoided the edge and showed higher abundance toward pastures. In the JF locality eight species were more abundant in the pasture, four of them with a complete response: *Canthon* (*Boreocanthon*) *puncticollis* LeConte, 1866, *Canthon humectus*, *Canthon imitator* Brown, 1946, and *Digitonthophagus gazella* Fabricius, 1787 (Fig. 2E), and four with an incomplete response within our sampled gradient (*Canthon cyanellus* LeConte, 1859, *Dichotomius colonicus* Say, 1835, *Glaphyrocanthon* sp., and *Phanaeus adonis*; Fig. 2F). Interestingly, in the POF locality four *Onthophagus* species avoided the edge and had higher abundance toward pastures: *O. mexicanus*, *O. incensus*, *O. knulli*, and *O. gibsoni* Howden & Génier 2004 (the first with a complete response, the second with an incomplete response, and the last two with undetermined responses).

Overall, the proportion of species showing different types of response to the edge effect was similar between localities (*G*_test_ = 2.84, *d.f.* = 2, *P* = 0.17, Table 2). Only eight species (40%) exhibited a complete response within the −90 to 90 m sampled transects, while for other 12 species the extent of edge effects was longer than this (40% with incomplete responses, 20% with undetermined responses), and these proportions remained similar in both forest types (*G*_test_ = 0.29, *d.f.* = 1, *P* = 0.63, Table 2).

### Assemblage responses to the edge

Contrary to our prediction, at the assemblage level dung beetle species richness, diversity, FRic, and FEve consistently had a neutral response to the edge, showing no clear preference for any habitat condition in both forest types (Table 1; Table S3). Abundance showed a neutral response in POF, but an avoidance response (incomplete) in the JF, where a higher number of individuals was captured toward pastures. On the contrary, FDiv responded neutrally in JF but had an avoidance incomplete response in POF, with higher values inside forest fragments (Table 1).

As expected, compositional dissimilarity showed a clear peak near the edge when species abundances were taken into account (1-Morisita index) in POF, while the Jaccard dissimilarity showed a neutral response to edge effects in the two forest types (Table 1).

## Discussion

How do dung beetles respond to edge effects in livestock dominated landscapes? In this work we detect different responses at the population level (a peak in abundance near the edge, edge avoidance, and edge insensitivity), and probably masked effects at the assemblage level due to the coexistence of species with different ecological requirements. Moreover, it seems that grazing maintains highly heterogeneous landscapes with a variety of microhabitats that enhances dung beetle diversity, although in other regions landscape heterogeneity has a negative effect on dung beetles (Lascaleia et al., 2018).

### Populations’ responses

Only *Copris incertus* showed a consistent pattern in both JF and POF (a neutral response to the edge). This big tunneler species is native from México, Central, and South America, and is common in tropical pasturelands (Cruz & Huerta, 1998), thus in our study area its abundance is not affected by the conversion of forests to pastures and can be considered as a habitat generalist.

Other five species had different responses to edge effects in the two studied forests, probably related to environmental changes due to their difference in vegetation structure, elevation (ca. 500 m), temperature (ca. 5 °C), and probably other variables. *Canthon humectus* and *Phanaeus adonis* have unimodal responses in POF, and an avoidance response with higher abundance in pastures in the JF locality. The biogeographic distribution pattern of these Neotropical species corresponds to the Plateau, and they have been reported in open and semi-open places usually with livestock (Morón, 2003; Halffter et al., 2011). *O. incensus* and *O. knulli* avoided the edge with higher abundance within forest in the *Juniperus* locality, but higher abundance in the pastures of POF; while *Onthophagus* sp. had a neutral response in JF but avoided the edge with higher abundance in the forest interior of POF. These *Onthophagus* species are widespread in the area, and near to our study area Barragán et al. (2014) found them more abundant on native vegetation than in pasturelands in a tropical forest landscape, however, on a pine-oak landscape, the same species are more abundant on pastures than in native vegetation. Several environmental variables associated with elevation and vegetation (such as soil hardness, humidity, temperature, slope, and tree cover) may have important effects on the responses of these species to environmental change. These interesting population responses to the environment should be further studied, probably at wider scales. The other 12 studied species had more than 30 individuals in only one of the two localities, thus we were not able to assess the consistency of their response patterns to edge effects.

In the same way as the habitat generalist *Copris incertus, Onthophagus* sp. in JF and *E. magnus* in POF also showed a neutral response to edge effects. Interestingly, *E. magnus* was likewise collected in both forest and savannah habitats in a sharp ecotone between open savannah and tall evergreen forest in Bolivia (Spector & Ayzama, 2003). Thus, this species behaves as a habitat generalist in these distant regions.

In six cases species avoided the edge and had greater abundance inside forest fragments, showing their sensibility to habitat conversion. Among them, only *O. igualensis* had a complete response within 90 m from the edge toward forest interior, while for the other species models showed that the extent of edge effects on abundance is larger than the sampled area (incomplete or undetermined responses). Our results indicate that all these species are characteristics of native forests in our studied area, so their habitat specificity becomes them vulnerable to habitat conversion and their ecological services might be compromised. For example, *Deltochilum scabriusculum* is one of the largest species in the area and as a result of its biomass it removes great amounts of dung in these forest ecosystems (Ortega-Martínez, Moreno & Escobar, 2016). Thus, forest conversion into open habitats may have strong effects on dung beetle functional groups and ecological services such as dung removal and seed post-dispersal (Hosaka et al., 2014).

In newly transformed pasturelands, native forest species are replaced by heat-tolerant species that endure the stressful conditions created in open, light filled agroecosystems (Halffter & Arellano, 2002). Thus, the low complexity of open-grazed areas, and the high availability of dung resources, may promote dominance of the species associated to open conditions habitats (Halffter, Favila & Arellano, 1995; Montes-De-Oca, 2001). In our study area eleven species avoided the edge and had higher abundances in pastures than in forest remnants. Heliophilous and thermophilous species such as *Canthon imitator* and *C*. (*B.*) *puncticollis* in JF, or *O. mexicanus* and *O. incensus* in POF, have abundant populations in pastures. The large *Dichotomius colonicus* was not very abundant but also avoided the edge and preferred the pasture. Similarly, in a mountain landscape of the MTZ *Dichotomius colonicus* was likewise abundant in pastures, and is supposed to use these agroecosystems as corridors to move from tropical landscapes to higher altitudes (Arellano & Halffter, 2003).

The exotic *Digitonthophagus gazella* is also abundant in pastures, especially in JF. This Asian–African beetle was introduced to America ca. 1972 and is now widespread in tropical and subtropical pastures (Montes-De-Oca & Halffter, 1998; Noriega, Moreno & Otavo, 2011). In the MTZ this species has invaded grazing habitats in lowlands and middle-mountain ecosystems (Barragán et al., 2014), probably because these pastures resemble its native habitats.

Regarding the extent of edge effects on populations, in almost half (11) of the 24 studied cases, species showed neutral, unimodal, or complete avoidance responses within the 90 m sampled at both sides of ecotones (Table 1). This coincides with the results of Spector & Ayzama (2003) in a forest-border-savannah system, where nearly half (24) of the species that were scored for habitat specificity were found in only one habitat, even when sampling was carried out only 50 m apart from the ecotones. Similarly, in forest–sun-grown coffee ecotones of Colombia Villada-Bedoya et al. (2017) found that the extent of some dung beetle species responses to edge effects occurred within 210 m from the ecotones. Furthermore, for ground beetles (Carabidae) clear edge effects have been detected in as near as 20 m from the ecotone (Boetzl, Schneider & Krauss, 2016; Jung & Lee, 2016). These examples show that many beetle populations might be sensitive to edge effects even at short distances, although this is not a generalized pattern. For example, Ewers & Didham (2008) found that the abundances of 20% of common beetle species caught in New Zealand were affected by edges at scales >250 m, and one in eight common species had edge effects that appeared to penetrate as far as one km into habitat patches. Therefore, large-scale edge effects may threaten forest interior species by promoting their local extinction in fragmented landscapes, and properly designed studies should been carried out to assess these processes.

Intrinsic characteristics of species, such as dispersal ability or tolerance to desiccation, determine dung beetle responses to habitat change (Montes-De-Oca, 2001; Vulinec et al., 2008). Thus, each species responds to edge effects in its own particular way, resulting in species-dependent filtration by edges (Ingham & Samways, 1996; Gaublomme, Eggermont & Hendrickx, 2014), or in spillover effects, that is, movements of species across the habitat edge due to contrasting resource availability in the adjacent habitats (Boetzl, Schneider & Krauss, 2016; Prass et al., 2017). These processes should be further studied at a landscape scale, accounting for the effects of broad scale factors that may influence beetles, such as the level of contrast between the native habitat and the matrix (Prass et al., 2017), overall landscape structure (Jung & Lee, 2016), or human activities in fragmented landscapes. For example, livestock produces different types of dung in variable quantities, thus resource availability may be a major driver of dung beetle abundance. In a recent study Bourg et al. (2016) found that tropical forest substitution by grasslands modifies the patterns of food selection by coprophagous beetles and affects the functioning of ecosystems. Therefore, we could expect that trophic generalist species will have neutral responses or will show unimodal preferences near to the edge, while food specialists will show avoidance to the edge responses (with species feeding on native mammal dung, rotten fruits or carrion being more abundant in forest interior, and species feeding in cattle dung being more abundant in pastures), as specialists are more sensitive to habitat fragmentation (Gaublomme, Eggermont & Hendrickx, 2014).

Our results with baited-pitfall traps show patterns on the abundance of species that fly to the food source. Some species may arrive from the surroundings to the ecotone for foraging, but their captures in traps do not indicate that they maintain viable populations on this area. Therefore, a future research line should explore which dung beetle species really nest at the ecotone. For example, in our study JF site, couples of *Canthon humectus* arrive to cattle dung, form a brood-ball and roll it 0.28–9.65 m away from the source until they bury it in pastureland (Ortega-Martínez et al., 2014). However, other species may not have a breeding population in pastures and our records in pitfall traps could be of individuals in transit (vagrants). Thus, the real habitat indicator character of species (forest of pasture preference) should be studied in deep with evidence of reproductive behavior.

### Assemblages’ responses

Despite differential responses of dung beetle populations to edge effects, at the assemblage level these effects are masked, as species richness, species diversity, FRic, and FEve did not vary along forest-pasture ecotones. Only total abundance had an avoidance response (incomplete) to the edge in the JF, where more individuals were captured in pastures.

This neutral response in assemblage parameters (or even a positive effect of habitat conversion on abundance) may depend strongly on the interaction of ecological factors and the biogeographical context (Davis et al., 2010). In the MTZ, other studies have also found that grazing areas have a positive effect on dung beetle abundance and diversity at high elevations and in dry environments, specifically in pine–oak forest and semiarid scrubland (Barragán et al., 2014) and JF (Ortega-Martínez, Moreno & Escobar, 2016) of the Sierra Madre Oriental province. Similarly, dung beetle abundance is higher in human-created pastures than in native savannah-like vegetation in the Cerrado ecosystem in Brazil, and the influence of the edge is evident only for abundance when landscape matrix is pastureland (Martello et al., 2016). Thus, the presence of cattle may have a positive effect on dung beetle abundance, probably due to higher resource availability (dung), but very intensive grazing may reduce their diversity (Hutton & Giller, 2003).

In contrast, in tropical forests habitat conversion to pastures does usually have a negative effect on dung beetle diversity (Nichols et al., 2007; Beiroz et al., 2017), and several empirical studies have shown that tropical native forests harbor more species and more abundance than pastures because dung beetles prefer shaded habitats than open areas where heat limits their behavior (Halffter & Arellano, 2002; Escobar, 2004; Vidaurre, Gonzales & Ledezma, 2008; Lee et al., 2009; Noriega et al., 2012). Besides higher temperatures, pastures face wind variations, which may be a limiting factor for some sensitive species (Halffter, 1991; Hanski & Cambefort, 1991; Basto-Estrella et al., 2012; Frank et al., 2017). However, edge effects not always have a clear influence on dung beetle assemblage parameters in tropical forests. For example, Spector & Ayzama (2003) found a strong effect on dung beetles species richness and biomass through forest-border-savanna, and Villada-Bedoya et al. (2017) also found higher richness and abundance in the forest when assessing forest-coffee ecotones, although species diversity was higher at the edge. In land use change studies dung beetle functional diversity is also impoverished in cattle pastures (Barragán et al., 2011; Gómez-Cifuentes et al., 2017). On the contrary, as shown in this study, in some cases the species richness of dung beetle assemblages is not sensitive to forest edge effects (Durães, Martins & Vaz-De-Mello, 2005; Feer, 2008).

In a similar way, the neutral response of FRic and FEve to edge effects may result from the balance between individuals and species lost and gained from one habitat to the adjacent. This balanced variation may result in forest and pasture assemblages that do not differ in their functional trait space (FRic), or in the regularity of abundance distribution in traits (FEve), regardless on the identity of species. The lack of change in the FEve of dung beetle assemblages also occurs when tropical forests are converted into cattle pastures (Barragán et al., 2011), thus FEve might not be related to environmental variables related to habitat change, but with the assembly mechanisms of ecological communities, such as niche filtering (Mouchet et al., 2010).

Functional divergence, which measures how abundance is distributed within the volume of functional trait space occupied by all the species, had higher values inside POF forest fragments. Therefore, high values within forests indicate that abundant species are distant from the center of gravity in the assemblage functional trait space, relative to rare species, as a result of dissimilarity in functional traits among species. Thus, the inclusion of functional diversity in our assemblage level of analysis brings about important insights into the mechanisms behind edge responses. Likewise, Magura (2017) found significant edge effects on ground beetle assemblages when functional, phylogenetic and functional-phylogenetic diversities were used across forest-grassland gradients. Indeed, an integral approach encompassing different dimensions of biological diversity is highly desirable. And, although identifying the influence of edge effects on particular functional traits or ecological processes regulated by dung beetles was beyond the scope of this study, we believe that edge effects on the functionality of ecosystems deserve additional research (Ziter, Bennett & Gonzalez, 2014). For example, it would be interesting to link the functional diversity of beetle assemblages to particular functional processes such as dung removal, seed dispersal and nutrient cycling not only in contrasting habitats, but along forest edges. Also, the relationships of functional diversity with specific environmental variables of ecotones remain unknown.

As expected, dung beetle compositional dissimilarity increased abruptly at the edge, fitting the unimodal function, at least in one forest. Certainly, dissimilarity in species composition changed without an apparent loss of species, as richness and functional diversity had a neutral response. Likewise, Ewers, Thorpe & Didham (2007) and Villada-Bedoya et al. (2017) proposed that the coexistence of species on the edge is more evident when diversity does not differ significantly between the adjacent components of the ecotone. Compositional dissimilarity was more evident when species abundances were taken into account, which corresponds with the results of abundance avoidance to the edge in most of the populations studied.

## Concluding remarks

Ecological research on edge effects has been intensive in natural ecotones, but in the last decades research has also focused on human-modified landscapes, where novel ecosystems are usually simple in structure and represent inhospitable habitats for many species. For example, Magura, Lövei & Tóthmérész (2017) found that natural ecotones have more species richness than ecotones created by human activities. Here we show different responses of dung beetle populations (a peak in abundance near the edge, edge avoidance, and edge insensitivity) in livestock dominated landscapes.

We encourage further studies at larger scales to assess other factors that may modulate edge effects, such as landscape composition and structure, metapopulation dispersal and metacommunity dynamics in fragmented landscapes, and the degree of contrast between habitats (Byers, Holmes & Malek, 2017). For example, Peyras et al. (2013) have shown that dung beetle species responses to edge effects depended on the environmental dissimilarity between habitats (low or high contrast ecotones).

Remnants of native forests are crucial to preserve all species at the landscape level (Beiroz et al., 2017), as several populations avoid the edge and maintain higher abundance toward forest interiors. Given the lack of large forest remnants and protected areas in our study region, small forest fragments within rangelands should be valued and their protection by local owners should be encouraged. Moreover, we would like to emphasize that conservation efforts in human-managed productive landscapes should focus on mitigating current and delayed edge effects in native ecosystem fragments, and on promoting sustainable management plans in livestock pastures. Particularly, we suggest creating buffer zones in pasture-native habitat ecotones and promoting landscape connectivity through wide forest corridors in order to reduce negative edge effects. Indeed, mixed responses of dung beetle populations and assemblages to edge effects suggest that these processes are important mechanisms affecting native species and assemblages. Therefore, ecotone and landscape management will be crucial in livestock dominated landscapes to allow regional conservation of biodiversity and the environmental services carried out by dung beetles.

## Supplemental Information

10.7717/peerj.6148/supp-1 Supplemental Information 1 Functional traits, biogeographic origin and abundance of dung beetle species collected in temperate forests of the Mexican Transition Zone. JF: *Juniperus* forest, POF: pine-oak forest. Food relocation (R-rollers, T-tunnelers, D-dwellers), Activity period (D-diurnal, N-nocturnal), diet type (C-coprophagous, C-N- copro-necrophagous). Biogeographic origin: NEO = Neotropical, HOL = Holarctic, AFR = Afrotropical. Biogeographical distribution pattern (BDP): PMo = Palaeoamerican Montane, PPl = Palaeoamerican Plateau, TPa = Tropical Palaeoamerican, MMo = Mesoamerican Montane, Pl = Plateau, NE = Neotropical.

10.7717/peerj.6148/supp-2 Supplemental Information 2 Dung beetle community parameters calculated for each sampling unit in *Juniperus* (JF) and pine-oak forest (POF) ecotones with pastures (twe sites per forest type). The ecotone sample is indicated with zero; distances of sampling points towards forest. Cn: sample coverage, FRic: functional richness, FEve: functional evenness, FDiv: functional divergence.

10.7717/peerj.6148/supp-3 Supplemental Information 3 Parameters of the model, or models, that best fit the data of species abundance along forest-pasture gradients in *Juniperus* (JF) and pine-oak forest (POF). n = number of dung beetle individuals collected. Undet. = undetermined.

10.7717/peerj.6148/supp-4 Supplemental Information 4 Number of dung beetles captured at each edge distance in the three transects per site. For the non-linear regression models we joined the number of individuals captured in the three transects for each site, as sampling units. Raw data.

## Additional Information and Declarations

### Data Availability

The following information was supplied regarding data availability: Raw data is provided in a Supplemental File.
