# Supplementary material for: Populations and assemblages living on the edge: dung beetles responses to forests-pasture ecotones
Source: PeerJ. 2018 Dec 13;6:e6148. doi: 10.7717/peerj.6148 (PMC6295328; doi:10.7717/peerj.6148)
Supplement: Supplemental Information 1 — Food relocation (R-rollers, T-tunnelers, D-dwellers), Activity period (D-diurnal, N-nocturnal), diet type (C-coprophagous, C-N- copro-necrophagous). Biogeographic origin: NEO = Neotropical, HOL = Holarctic, AFR = Afrotropical. Biogeographical distribution pattern (BDP): PMo = Palaeoamerican Montane, PPl = Palaeoamerican Plateau, TPa = Tropical Palaeoamerican, MMo = Mesoamerican Montane, Pl = Plateau, NE = Neotropical. [file peerj-06-6148-s001.doc]

| **Species** | **Food relocation** | **Activity period** | **Diet type** | **Biomass (mg)** | **Biogeographic origin** | **BDP** | **Abundance (POF)** | **Abundance (JF)** |
| --- | --- | --- | --- | --- | --- | --- | --- | --- |
| *Canthon cyanellus cyanellus* LeConte, 1859 | R | D | C-N | 23 | NEO | NE | 0 | 47 |
| *Canthon (Canthon) humectus hidalgoensis* Bates, 1887 | R | D | C-N | 55 | NEO | Pl | 173 | 9729 |
| *Canthon (Canthon) imitator* Brown, 1946 | R | D | C-N | 79 | NEO | ? | 6 | 1764 |
| *Canthon (Boreocanthon) puncticollis* LeConte, 1866 | R | D | C | 8 | NEO | NE | 0 | 768 |
| *Copris incertus* Say, 1835 | T | N | C-N | 91 | HOL | PPl | 40 | 52 |
| *Copris klugi* Harold, 1869 | T | N | C-N | 121 | HOL | PMo | 20 | 0 |
| *Copris lugubris* Boheman, 1858 | T | N | C-N | 124 | HOL | ? | 14 | 0 |
| *Deltochilum scabriusculum* Bates, 1887 | R | N | C-N | 253 | NEO | NE | 1 | 31 |
| *Dichotomius colonicus* (Say, 1835) | T | N | C | 346 | NEO | Pl | 12 | 32 |
| *Digitonthophagus gazella* (Fabricius, 1787) | T | D | C | 24 | AFRO | EXO | 0 | 2118 |
| *Euoniticellus intermedius* (Reiche, 1849) | T | D | C | 10 | AFR | EXO | 2 | 5 |
| *Eurysternus magnus* Laporte de Castelnau, 1840 | D | D | C-N | 70 | NEO | Mmo | 29 | 7 |
| *Glaphyrocanthon* sp. | R | D | C-N | 5 | NEO | NE | 0 | 58 |
| *Onthophagus gibsoni* Howden & Génier 2004 | T | N | C | 6 | HOL | PPl | 185 | 3 |
| *Onthophagus igualensis* Bates, 1887 | T | D | C-N | 5 | HOL | PPl | 4 | 38 |
| *Onthophagus incensus* Say, 1835 | T | D | C-N | 15 | HOL | Mmo | 2284 | 8534 |
| *Onthophagus knulli* Howden & Cartwright, 1963 | T | D | C-N | 3 | HOL | ? | 418 | 6350 |
| *Onthophagus mexicanus* Bates, 1887 | T | D | C-N | 12 | HOL | PPl | 4356 | 10 |
| *Onthophagus* sp. | T | D | C-N | 4 | HOL | ? | 155 | 283 |
| *Phanaeus (Phanaeus) adonis* Harold, 1863 | T | D | C-N | 110 | NEO | Pl | 506 | 780 |
| *Pseudocanthon chlorizans* Bates, 1887 | R | D | C | 4 | NEO | NE | 0 | 26 |
| *Sysiphus mexicanus* Harold, 1863 | R | D | C-N | 19 | AFR-HOL | ? | 0 | 2796 |
