# Supplementary material for: Populations and assemblages living on the edge: dung beetles responses to forests-pasture ecotones
Source: PeerJ. 2018 Dec 13;6:e6148. doi: 10.7717/peerj.6148 (PMC6295328; doi:10.7717/peerj.6148)
Supplement: Supplemental Information 2 — Cn: sample coverage, FRic: functional richness, FEve: functional evenness, FDiv: functional divergence. [file peerj-06-6148-s002.docx]

| **Sampling unit** | **Species richness** | **Abundance** | **C*n*** | **Diversity *q*=1** | **FRic** | **FEve** | **FDiv** | **Jaccard dissimilarity** | **Morisita dissimilarity** |
| --- | --- | --- | --- | --- | --- | --- | --- | --- | --- |
| POF1_90 | 11 | 1096 | 0.9982 | 2.94 | 6.63 | 0.39 | 0.79 | 0.2500 | 0.0079 |
| POF1_60 | 10 | 1025 | 0.9990 | 3.51 | 11.88 | 0.23 | 0.80 | 0.3250 | 0.1052 |
| POF1_30 | 14 | 817 | 0.9951 | 4.91 | 12.44 | 0.23 | 0.93 | 0.3429 | 0.1108 |
| POF1_0 | 10 | 395 | 0.9949 | 4.54 | 7.16 | 0.35 | 0.86 | 0.2582 | 0.0198 |
| POF1_-30 | 13 | 332 | 0.9940 | 5.44 | 7.46 | 0.34 | 0.83 | 0.2308 | 0.0782 |
| POF1_-60 | 10 | 195 | 0.9898 | 3.22 | 7.08 | 0.30 | 0.87 | 0.2972 | 0.0770 |
| POF1_-90 | 8 | 166 | 0.9940 | 3.28 | 1.35 | 0.34 | 0.67 | 0.3636 | 0.0181 |
| POF2_90 | 12 | 647 | 0.9954 | 3.68 | 7.08 | 0.47 | 0.85 | 0.3846 | 0.0820 |
| POF2_60 | 9 | 1374 | 0.9993 | 2.36 | 1.65 | 0.40 | 0.65 | 0.3846 | 0.0414 |
| POF2_30 | 12 | 1326 | 0.9977 | 2.39 | 1.62 | 0.41 | 0.62 | 0.4066 | 0.1820 |
| POF2_0 | 10 | 346 | 0.9971 | 4.52 | 1.95 | 0.44 | 0.71 | 0.2643 | 0.2616 |
| POF2_-30 | 9 | 195 | 0.9897 | 2.79 | 1.76 | 0.34 | 0.79 | 0.1500 | 0.0976 |
| POF2_-60 | 9 | 142 | 1.0000 | 3.73 | 2.28 | 0.22 | 0.78 | 0.1000 | 0.0200 |
| POF2_-90 | 9 | 149 | 0.9868 | 3.52 | 2.28 | 0.23 | 0.79 | 0.0000 | 0.0048 |
| JF1_90 | 14 | 2602 | 0.9992 | 4.20 | 13.77 | 0.22 | 0.77 | 0.1333 | 0.0047 |
| JF1_60 | 14 | 3284 | 0.9994 | 4.51 | 13.88 | 0.11 | 0.81 | 0.2333 | 0.0030 |
| JF1_30 | 16 | 2764 | 0.9989 | 4.83 | 13.46 | 0.19 | 0.73 | 0.3611 | 0.3387 |
| JF1_0 | 13 | 1533 | 0.9994 | 4.38 | 21.39 | 0.27 | 0.80 | 0.3819 | 0.3800 |
| JF1_-30 | 13 | 832 | 1.0000 | 5.23 | 12.32 | 0.37 | 0.82 | 0.3875 | 0.0797 |
| JF1_-60 | 11 | 898 | 0.9978 | 5.23 | 2.94 | 0.17 | 0.75 | 0.3071 | 0.0579 |
| JF1_-90 | 14 | 1353 | 0.9985 | 5.31 | 5.65 | 0.22 | 0.84 | 0.2143 | 0.0400 |
| JF2_90 | 14 | 2278 | 0.9991 | 5.22 | 13.27 | 0.39 | 0.72 | 0.3125 | 0.0037 |
| JF2_60 | 13 | 2267 | 0.9996 | 5.62 | 3.55 | 0.42 | 0.79 | 0.2563 | 0.0217 |
| JF2_30 | 14 | 3260 | 0.9994 | 5.30 | 7.76 | 0.10 | 0.72 | 0.2389 | 0.1749 |
| JF2_0 | 17 | 3292 | 0.9994 | 4.39 | 18.32 | 0.28 | 0.89 | 0.2500 | 0.1605 |
| JF2_-30 | 15 | 3002 | 0.9993 | 4.07 | 12.20 | 0.21 | 0.85 | 0.2049 | 0.0144 |
| JF2_-60 | 14 | 2461 | 0.9992 | 3.90 | 5.65 | 0.27 | 0.94 | 0.2009 | 0.0170 |
| JF2_-90 | 11 | 3605 | 0.9997 | 3.68 | 5.65 | 0.32 | 0.93 | 0.2143 | 0.0158 |
