# Supplementary material for: Populations and assemblages living on the edge: dung beetles responses to forests-pasture ecotones
Source: PeerJ. 2018 Dec 13;6:e6148. doi: 10.7717/peerj.6148 (PMC6295328; doi:10.7717/peerj.6148)
Supplement: Supplemental Information 3 — n = number of dung beetle individuals collected. Undet. = undetermined. [file peerj-06-6148-s003.docx]

|  |  | **JF** | | | **POF** | | |
| --- | --- | --- | --- | --- | --- | --- | --- |
| **Species / assemblage parameter** | **Combined** | **n** | **Best model** | **Parameters** | **n** | **Best model** | **Parameters** |
| *Canthon cyanellus cyanellus* LeConte, 1859 | Linear  Power  (Incomplete) | 47 | Linear | b = 0.59  Yo = 0.005 | 0 | -- | -- |
|  |  |  | Power | Yo = 0.53  k = 0.0089 |  |  |  |
| *Canthon humectus hidalgoensis* Bates, 1887 | Sigmoid  Unimodal  (Complete) | 9729 | Sigmoid | Ymin = 0.25  Ymax = 1.06  B2 = 4.89  B3 = 0.48 | 143 | Unimodal | Ymin = 0.00  Ymax= 1.32  B2 = 95.5  B3 = 0.06  B4 = 0.01 |
| *Canthon imitator* Brown, 1946 |  | 1674 | Sigmoid | Ymax =  B2 =  B3 = | 5 | -- | -- |
| *Canthon (Boreocanthon) puncticollis* LeConte, 1866 | Sigmoid  (Complete) | 768 | Sigmoid | Ymin = 0.00  Ymax = 1.27  B2 = 28.2  B3 = 0.44 |  | -- | -- |
| *Copris incertus* Say, 1835 | Neutral | 52 | Mean | a = 0.59 | 39 | Mean | a = 0.60 |
| *Copris klugi* Harold, 1869 | -- | 0 | -- | -- | 19 | -- | -- |
| *Copris lugubris* Boheman, 1858 | -- | 0 | -- | -- | 11 | -- | -- |
| *Deltochilum scabriusculum* Bates, 1887 | Linear  Sigmoid  (Undet.) | 31 | Linear | b = -0.007  Yo = 0.54 | 1 | -- | -- |
|  |  |  | Sigmoid | Ymin = 0.02  Ymax = 1.00  B2 = 7.49  B3 = -0.11 |  |  |  |
| *Dichotomius colonicus* (Say, 1835) | Linear  Power  (Incomplete) | 32 | Linear | b = 0.003  Yo = 0.63 | 12 | -- | -- |
|  |  |  | Power | Yo = 0.61  k = 0.005  Tau = 197 |  |  |  |
| *Digitonthophagus gazella* (Fabricius, 1787) | Sigmoid  (Complete) | 2118 | Sigmoid | Ymin = 0.01  Ymax= 1.24  B2 = 17.6  B3 = 0.13 | 0 | -- | -- |
| *Euoniticellus intermedius* (Reiche, 1849) | -- | 5 | -- | -- | 2 | -- | -- |
| *Eurysternus magnus* Laporte de Castelnau, 1840 | Neutral | 7 | -- | --- | 31 | Mean | a = 0.60 |
| *Glaphyrocanthon* sp. | Linear  Power  (Incomplete) | 58 | Linear | b = -0.006  Yo = 0.59 | 0 | -- | -- |
|  |  |  | Power | Yo = 0.50  k = -0.01  Tau = -95 |  |  |  |
| *Onthophagus gibsoni* Howden & Génier 2004 | Linear  Sigmoid  (Undet.) | 3 | -- | -- | 177 | Linear | b = 0.007  Yo = 0.57 |
|  |  |  |  |  |  | Sigmoid | Ymin = 0.07  Ymax = 1.08  B2 = 0.37  B3 = 0.08 |
| *Onthophagus incensus* Say, 1835 | Linear  Power  (Incomplete) | 8534 | Linear | b = 0.00086  Yo = 0.052 | 2190 | Linear | b = 0.003  Yo = 0.67 |
|  |  |  | Power | Yo = 0.66  k = -0.003 |  | Power | Yo = 0.55  k = 0.0038 |
| *Onthophagus igualensis* Bates, 1887 | Sigmoid  (Complete) | 38 | Sigmoid | Ymin = 0.00  Ymax = 1.35  B2 = -27.3  B3 = -0.011 | 4 | -- | -- |
| *Onthophagus knulli* Howden & Cartwright, 1963 | Linear  Sigmoid  (Undet.) | 6350 | Linear | b = - 0.0039  Yo = 0.63 | 409 | Linear | b = 0.006  Yo = 0.62 |
|  |  |  | Sigmoid | Ymin = 0.22  Ymax = 0.87  B2 = 29.9  B3 = 10.84 |  | Sigmoid | Ymin = 0.12  Ymax= 1.04  B2 = -10.1  B3 = 0.06 |
| *Onthophagus mexicanus* Bates, 1887 | Sigmoid  (Complete) | 10 | -- | -- | 4304 | Sigmoid | Ymin = 0.06  Ymax = 1.15  B2 = 11.33  B3 = 0.11 |
| *Onthophagus* sp. | Neutral | 283 | Mean | a = 0.57 | 155 | Linear | b = -0.003  Yo = 0.64 |
|  |  |  |  |  |  | Power | Yo = 0.062  k = -0.005 |
| *Phanaeus (Phanaeus) adonis* Harold, 1863 | Linear  Power  Unimodal  (Undet.) | 780 | Linear | b = 0.004  Yo = 0.63 | 460 | Unimodal | Ymin = 0.07  Ymax = 1.33  B2 = -2.94  B3 = 0.08  B4 = 0.01 |
|  |  |  | Power | Yo = 0.59  k = 0.006  Tau = 153 |  |  |  |
| *Sysiphus mexicanus* Harold, 1863 | Linear  (Incomplete) | 2796 | Linear | b = -0.0069  Yo = 0.60 | 0 | -- | -- |
| *Pseudocanthon chlorizans* Bates, 1887 | -- | 26 | -- | -- | 0 | -- | -- |
| **Assemblages** | | | | | | | |
| Species richness | Neutral |  | Mean | a = 9.4-11.4 |  | Mean | a = 12.8-14.7 |
| Abundance | Neutral |  | Mean | a = 322-849 |  | Mean | a = 1859-2917 |
| Diversity | Neutral |  | Mean | a = 3.1-4.2 |  | Mean | a = 4.3-5.1 |
| FRic | Neutral |  | Mean | a = 2.9-7.4 |  | Mean | a = 7.4-13.9 |
| FEve | Neutral |  | Mean | a = 0.29-0.38 |  | Mean | a = 0.19-0.31 |
| FDiv | Neutral  Linear  Power  (Undet.) |  | Mean | a =0.73-0.83 |  | Linear | b = -0.0007  Yo = 0.81 |
|  |  |  |  |  |  | Power | Yo = 0.81  k = -0.0009 |
| Jaccard dissimilarity | Linear,  Power  (Incomplete) |  | Linear | a = -0.001  Yo = 0.72 |  | Power | Yo = 0.63  k = -0.004 |
|  |  |  | Power | Yo = 0.71  k = -0.0027 |  |  |  |
| Morisita dissimilarity | Linear  Power  Sigmoid  (Undeter.) |  | Linear | a = 0.77  Yo = -0.003 |  | Sigmoid | Ymin = 0.47  Ymax = 0.98  B2 = 18.2  B3 = -0.10 |
|  |  |  | Power | Yo = 0.75  k = -0.004  Tau = -246 |  |  |  |
