# Supplementary material for: Populations and assemblages living on the edge: dung beetles responses to forests-pasture ecotones
Source: PeerJ. 2018 Dec 13;6:e6148. doi: 10.7717/peerj.6148 (PMC6295328; doi:10.7717/peerj.6148)
Supplement: Supplemental Information 4 — Raw data. [file peerj-06-6148-s004.docx]

|  | | | | **Edge distance (m)** | | | | | | |  |
| --- | --- | --- | --- | --- | --- | --- | --- | --- | --- | --- | --- |
|  |  |  |  | **Forest** | | | **Edge** | **Pasture** | | |  |
| **Species** | **Forest** | **Site** | **Transect** | **-90** | **-60** | **-30** | **0** | **30** | **60** | **90** | **Total** |
| *Boreocanthon puncticollis* | JF | 1 | 1 | 0 | 0 | 0 | 0 | 26 | 77 | 49 | 152 |
|  |  |  | 2 | 0 | 0 | 0 | 0 | 120 | 58 | 23 | 201 |
|  |  |  | 3 | 0 | 0 | 0 | 0 | 59 | 66 | 38 | 163 |
|  |  | 2 | 1 | 0 | 0 | 0 | 0 | 5 | 23 | 46 | 74 |
|  |  |  | 2 | 0 | 0 | 0 | 1 | 11 | 49 | 52 | 113 |
|  |  |  | 3 | 0 | 0 | 0 | 0 | 3 | 45 | 17 | 65 |
|  | POF | 1 | 1 | 0 | 0 | 0 | 0 | 0 | 0 | 0 | 0 |
|  |  |  | 2 | 0 | 0 | 0 | 0 | 0 | 0 | 0 | 0 |
|  |  |  | 3 | 0 | 0 | 0 | 0 | 0 | 0 | 0 | 0 |
|  |  | 2 | 1 | 0 | 0 | 0 | 0 | 0 | 0 | 0 | 0 |
|  |  |  | 2 | 0 | 0 | 0 | 0 | 0 | 0 | 0 | 0 |
|  |  |  | 3 | 0 | 0 | 0 | 0 | 0 | 0 | 0 | 0 |
| *Canthon cyanellus* | JF | 1 | 1 | 0 | 0 | 2 | 0 | 1 | 1 | 1 | 5 |
|  |  |  | 2 | 2 | 0 | 0 | 0 | 0 | 0 | 1 | 3 |
|  |  |  | 3 | 0 | 0 | 1 | 0 | 0 | 4 | 3 | 8 |
|  |  | 2 | 1 | 0 | 0 | 0 | 1 | 3 | 1 | 1 | 6 |
|  |  |  | 2 | 0 | 1 | 0 | 1 | 4 | 5 | 2 | 13 |
|  |  |  | 3 | 0 | 0 | 1 | 1 | 1 | 6 | 3 | 12 |
|  | POF | 1 | 1 | 0 | 0 | 0 | 0 | 0 | 0 | 0 | 0 |
|  |  |  | 2 | 0 | 0 | 0 | 0 | 0 | 0 | 0 | 0 |
|  |  |  | 3 | 0 | 0 | 0 | 0 | 0 | 0 | 0 | 0 |
|  |  | 2 | 1 | 0 | 0 | 0 | 0 | 0 | 0 | 0 | 0 |
|  |  |  | 2 | 0 | 0 | 0 | 0 | 0 | 0 | 0 | 0 |
|  |  |  | 3 | 0 | 0 | 0 | 0 | 0 | 0 | 0 | 0 |
| *Canthon humectus hidalgoensis* | JF | 1 | 1 | 122 | 274 | 125 | 0 | 475 | 494 | 566 | 2056 |
|  |  |  | 2 | 146 | 1 | 1 | 92 | 576 | 557 | 464 | 1837 |
|  |  |  | 3 | 61 | 35 | 41 | 34 | 326 | 623 | 400 | 1520 |
|  |  | 2 | 1 | 65 | 19 | 17 | 100 | 563 | 214 | 420 | 1398 |
|  |  |  | 2 | 44 | 44 | 73 | 188 | 546 | 521 | 305 | 1721 |
|  |  |  | 3 | 48 | 33 | 101 | 110 | 272 | 277 | 356 | 1197 |
|  | POF | 1 | 1 | 0 | 0 | 0 | 0 | 1 | 3 | 3 | 7 |
|  |  |  | 2 | 0 | 0 | 0 | 2 | 6 | 15 | 20 | 43 |
|  |  |  | 3 | 0 | 1 | 30 | 8 | 52 | 8 | 1 | 100 |
|  |  | 2 | 1 | 0 | 0 | 0 | 1 | 3 | 4 | 0 | 8 |
|  |  |  | 2 | 0 | 0 | 0 | 0 | 1 | 0 | 0 | 1 |
|  |  |  | 3 | 0 | 0 | 0 | 0 | 6 | 8 | 0 | 14 |
| *Canthon imitator* | JF | 1 | 1 | 10 | 43 | 30 | 0 | 71 | 127 | 85 | 366 |
|  |  |  | 2 | 27 | 0 | 0 | 25 | 119 | 99 | 97 | 367 |
|  |  |  | 3 | 4 | 16 | 4 | 0 | 66 | 90 | 61 | 241 |
|  |  | 2 | 1 | 0 | 1 | 3 | 3 | 97 | 44 | 90 | 238 |
|  |  |  | 2 | 5 | 2 | 10 | 16 | 102 | 97 | 62 | 294 |
|  |  |  | 3 | 2 | 3 | 9 | 11 | 44 | 85 | 104 | 258 |
|  | POF | 1 | 1 | 0 | 0 | 0 | 0 | 0 | 0 | 0 | 0 |
|  |  |  | 2 | 0 | 0 | 0 | 0 | 0 | 0 | 1 | 1 |
|  |  |  | 3 | 0 | 0 | 1 | 0 | 1 | 0 | 0 | 2 |
|  |  | 2 | 1 | 0 | 0 | 0 | 0 | 0 | 0 | 0 | 0 |
|  |  |  | 2 | 0 | 0 | 0 | 0 | 0 | 0 | 1 | 1 |
|  |  |  | 3 | 0 | 0 | 0 | 0 | 1 | 1 | 0 | 2 |
| *Copris incertus* | JF | 1 | 1 | 0 | 0 | 0 | 1 | 0 | 0 | 0 | 1 |
|  |  |  | 2 | 0 | 0 | 4 | 2 | 2 | 0 | 1 | 9 |
|  |  |  | 3 | 2 | 0 | 0 | 2 | 4 | 0 | 0 | 8 |
|  |  | 2 | 1 | 4 | 0 | 0 | 4 | 0 | 0 | 1 | 9 |
|  |  |  | 2 | 3 | 4 | 7 | 0 | 0 | 0 | 0 | 14 |
|  |  |  | 3 | 2 | 3 | 1 | 5 | 0 | 0 | 0 | 11 |
|  | POF | 1 | 1 | 2 | 0 | 1 | 2 | 2 | 0 | 2 | 9 |
|  |  |  | 2 | 1 | 2 | 0 | 0 | 2 | 0 | 0 | 5 |
|  |  |  | 3 | 0 | 0 | 1 | 1 | 0 | 0 | 0 | 2 |
|  |  | 2 | 1 | 1 | 0 | 1 | 3 | 0 | 1 | 0 | 6 |
|  |  |  | 2 | 1 | 1 | 0 | 3 | 0 | 2 | 1 | 8 |
|  |  |  | 3 | 0 | 1 | 2 | 1 | 0 | 3 | 3 | 10 |
| *Copris klugi* | JF | 1 | 1 | 0 | 0 | 0 | 0 | 0 | 0 | 0 | 0 |
|  |  |  | 2 | 0 | 0 | 0 | 0 | 0 | 0 | 0 | 0 |
|  |  |  | 3 | 0 | 0 | 0 | 0 | 0 | 0 | 0 | 0 |
|  |  | 2 | 1 | 0 | 0 | 0 | 0 | 0 | 0 | 0 | 0 |
|  |  |  | 2 | 0 | 0 | 0 | 0 | 0 | 0 | 0 | 0 |
|  |  |  | 3 | 0 | 0 | 0 | 0 | 0 | 0 | 0 | 0 |
|  | POF | 1 | 1 | 0 | 0 | 0 | 0 | 1 | 0 | 0 | 1 |
|  |  |  | 2 | 0 | 0 | 0 | 1 | 0 | 0 | 0 | 1 |
|  |  |  | 3 | 0 | 0 | 1 | 0 | 0 | 0 | 0 | 1 |
|  |  | 2 | 1 | 2 | 0 | 1 | 1 | 0 | 0 | 0 | 4 |
|  |  |  | 2 | 2 | 2 | 0 | 1 | 0 | 0 | 3 | 8 |
|  |  |  | 3 | 1 | 4 | 0 | 0 | 0 | 0 | 0 | 5 |
| *Copris lugubris* | JF | 1 | 1 | 0 | 0 | 0 | 0 | 0 | 0 | 0 | 0 |
|  |  |  | 2 | 0 | 0 | 0 | 0 | 0 | 0 | 0 | 0 |
|  |  |  | 3 | 0 | 0 | 0 | 0 | 0 | 0 | 0 | 0 |
|  |  | 2 | 1 | 0 | 0 | 0 | 0 | 0 | 0 | 0 | 0 |
|  |  |  | 2 | 0 | 0 | 0 | 0 | 0 | 0 | 0 | 0 |
|  |  |  | 3 | 0 | 0 | 0 | 0 | 0 | 0 | 0 | 0 |
|  | POF | 1 | 1 | 0 | 0 | 0 | 0 | 1 | 0 | 0 | 1 |
|  |  |  | 2 | 0 | 2 | 1 | 0 | 0 | 0 | 0 | 3 |
|  |  |  | 3 | 0 | 0 | 2 | 0 | 0 | 0 | 0 | 2 |
|  |  | 2 | 1 | 1 | 0 | 0 | 0 | 0 | 0 | 0 | 1 |
|  |  |  | 2 | 1 | 0 | 0 | 0 | 1 | 0 | 1 | 3 |
|  |  |  | 3 | 0 | 4 | 0 | 0 | 0 | 0 | 0 | 4 |
| *Deltochilum scabriusculum* | JF | 1 | 1 | 0 | 1 | 3 | 2 | 0 | 0 | 0 | 6 |
|  |  |  | 2 | 0 | 1 | 1 | 0 | 0 | 0 | 0 | 2 |
|  |  |  | 3 | 1 | 0 | 2 | 0 | 0 | 0 | 0 | 3 |
|  |  | 2 | 1 | 4 | 0 | 2 | 0 | 1 | 0 | 0 | 7 |
|  |  |  | 2 | 2 | 0 | 1 | 1 | 0 | 0 | 0 | 4 |
|  |  |  | 3 | 3 | 3 | 2 | 1 | 0 | 0 | 0 | 9 |
|  | POF | 1 | 1 | 0 | 0 | 0 | 0 | 0 | 0 | 0 | 0 |
|  |  |  | 2 | 0 | 0 | 0 | 0 | 0 | 0 | 0 | 0 |
|  |  |  | 3 | 0 | 0 | 0 | 0 | 1 | 0 | 0 | 1 |
|  |  | 2 | 1 | 0 | 0 | 0 | 0 | 0 | 0 | 0 | 0 |
|  |  |  | 2 | 0 | 0 | 0 | 0 | 0 | 0 | 0 | 0 |
|  |  |  | 3 | 0 | 0 | 0 | 0 | 0 | 0 | 0 | 0 |
| *Dichotomius colonicus* | JF | 1 | 1 | 0 | 0 | 0 | 1 | 1 | 0 | 0 | 2 |
|  |  |  | 2 | 0 | 0 | 0 | 0 | 0 | 0 | 2 | 2 |
|  |  |  | 3 | 0 | 0 | 1 | 1 | 0 | 1 | 0 | 3 |
|  |  | 2 | 1 | 1 | 0 | 0 | 1 | 0 | 0 | 2 | 4 |
|  |  |  | 2 | 0 | 2 | 2 | 1 | 1 | 1 | 2 | 9 |
|  |  |  | 3 | 0 | 2 | 3 | 2 | 2 | 0 | 3 | 12 |
|  | POF | 1 | 1 | 0 | 0 | 1 | 0 | 1 | 0 | 0 | 2 |
|  |  |  | 2 | 0 | 1 | 0 | 1 | 1 | 1 | 1 | 5 |
|  |  |  | 3 | 0 | 2 | 1 | 0 | 0 | 1 | 0 | 4 |
|  |  | 2 | 1 | 0 | 0 | 0 | 0 | 0 | 0 | 0 | 0 |
|  |  |  | 2 | 0 | 0 | 0 | 0 | 0 | 0 | 1 | 1 |
|  |  |  | 3 | 0 | 0 | 0 | 0 | 0 | 0 | 0 | 0 |
| *Digitonthophagus gazella* | JF | 1 | 1 | 0 | 0 | 0 | 0 | 88 | 212 | 149 | 449 |
|  |  |  | 2 | 0 | 0 | 0 | 3 | 135 | 152 | 65 | 355 |
|  |  |  | 3 | 0 | 0 | 0 | 3 | 241 | 227 | 175 | 646 |
|  |  | 2 | 1 | 0 | 0 | 0 | 5 | 13 | 42 | 142 | 202 |
|  |  |  | 2 | 0 | 0 | 3 | 14 | 52 | 108 | 49 | 226 |
|  |  |  | 3 | 0 | 0 | 1 | 2 | 78 | 88 | 71 | 240 |
|  | POF | 1 | 1 | 0 | 0 | 0 | 0 | 0 | 0 | 0 | 0 |
|  |  |  | 2 | 0 | 0 | 0 | 0 | 0 | 0 | 0 | 0 |
|  |  |  | 3 | 0 | 0 | 0 | 0 | 0 | 0 | 0 | 0 |
|  |  | 2 | 1 | 0 | 0 | 0 | 0 | 0 | 0 | 0 | 0 |
|  |  |  | 2 | 0 | 0 | 0 | 0 | 0 | 0 | 0 | 0 |
|  |  |  | 3 | 0 | 0 | 0 | 0 | 0 | 0 | 0 | 0 |
| *Euoniticellus intermedius* | JF | 1 | 1 | 0 | 0 | 0 | 0 | 0 | 0 | 0 | 0 |
|  |  |  | 2 | 0 | 0 | 0 | 0 | 1 | 0 | 0 | 1 |
|  |  |  | 3 | 0 | 0 | 0 | 0 | 1 | 0 | 0 | 1 |
|  |  | 2 | 1 | 0 | 0 | 0 | 0 | 0 | 0 | 1 | 1 |
|  |  |  | 2 | 0 | 0 | 0 | 0 | 1 | 0 | 0 | 1 |
|  |  |  | 3 | 0 | 0 | 0 | 0 | 0 | 0 | 1 | 1 |
|  | POF | 1 | 1 | 0 | 0 | 0 | 0 | 0 | 0 | 0 | 0 |
|  |  |  | 2 | 0 | 0 | 0 | 0 | 0 | 0 | 0 | 0 |
|  |  |  | 3 | 0 | 0 | 0 | 0 | 0 | 0 | 0 | 0 |
|  |  | 2 | 1 | 0 | 0 | 0 | 0 | 0 | 0 | 0 | 0 |
|  |  |  | 2 | 0 | 0 | 0 | 0 | 0 | 0 | 0 | 0 |
|  |  |  | 3 | 0 | 0 | 0 | 0 | 2 | 0 | 0 | 2 |
| *Eurysternus magnus* | JF | 1 | 1 | 1 | 1 | 0 | 0 | 0 | 0 | 0 | 2 |
|  |  |  | 2 | 1 | 0 | 0 | 0 | 1 | 0 | 0 | 2 |
|  |  |  | 3 | 0 | 0 | 0 | 0 | 0 | 0 | 0 | 0 |
|  |  | 2 | 1 | 0 | 0 | 0 | 2 | 0 | 0 | 0 | 2 |
|  |  |  | 2 | 0 | 0 | 1 | 0 | 0 | 0 | 0 | 1 |
|  |  |  | 3 | 0 | 0 | 0 | 0 | 0 | 0 | 0 | 0 |
|  | POF | 1 | 1 | 1 | 0 | 0 | 0 | 1 | 1 | 2 | 5 |
|  |  |  | 2 | 0 | 0 | 2 | 0 | 1 | 1 | 0 | 4 |
|  |  |  | 3 | 0 | 0 | 0 | 0 | 0 | 1 | 1 | 2 |
|  |  | 2 | 1 | 7 | 0 | 1 | 2 | 1 | 0 | 2 | 13 |
|  |  |  | 2 | 0 | 0 | 0 | 1 | 0 | 0 | 1 | 2 |
|  |  |  | 3 | 1 | 2 | 0 | 0 | 0 | 0 | 0 | 3 |
| *Glaphyrocanthon sp.* | JF | 1 | 1 | 6 | 8 | 2 | 0 | 0 | 1 | 1 | 18 |
|  |  |  | 2 | 4 | 0 | 2 | 2 | 1 | 0 | 0 | 9 |
|  |  |  | 3 | 6 | 7 | 0 | 0 | 1 | 1 | 0 | 15 |
|  |  | 2 | 1 | 0 | 4 | 0 | 2 | 0 | 0 | 0 | 6 |
|  |  |  | 2 | 3 | 0 | 2 | 1 | 0 | 0 | 0 | 6 |
|  |  |  | 3 | 1 | 0 | 1 | 1 | 0 | 0 | 1 | 4 |
|  | POF | 1 | 1 | 0 | 0 | 0 | 0 | 0 | 0 | 0 | 0 |
|  |  |  | 2 | 0 | 0 | 0 | 0 | 0 | 0 | 0 | 0 |
|  |  |  | 3 | 0 | 0 | 0 | 0 | 0 | 0 | 0 | 0 |
|  |  | 2 | 1 | 0 | 0 | 0 | 0 | 0 | 0 | 0 | 0 |
|  |  |  | 2 | 0 | 0 | 0 | 0 | 0 | 0 | 0 | 0 |
|  |  |  | 3 | 0 | 0 | 0 | 0 | 0 | 0 | 0 | 0 |
| *Onthophagus gibsoni* | JF | 1 | 1 | 1 | 0 | 0 | 0 | 0 | 1 | 0 | 2 |
|  |  |  | 2 | 0 | 0 | 0 | 0 | 0 | 0 | 0 | 0 |
|  |  |  | 3 | 0 | 0 | 0 | 1 | 0 | 0 | 0 | 1 |
|  |  | 2 | 1 | 0 | 0 | 0 | 0 | 0 | 0 | 0 | 0 |
|  |  |  | 2 | 0 | 0 | 0 | 0 | 0 | 0 | 0 | 0 |
|  |  |  | 3 | 0 | 0 | 0 | 0 | 0 | 0 | 0 | 0 |
|  | POF | 1 | 1 | 0 | 0 | 5 | 4 | 1 | 22 | 8 | 40 |
|  |  |  | 2 | 0 | 1 | 1 | 2 | 0 | 4 | 6 | 14 |
|  |  |  | 3 | 3 | 0 | 2 | 4 | 9 | 7 | 0 | 25 |
|  |  | 2 | 1 | 0 | 0 | 1 | 2 | 9 | 18 | 9 | 39 |
|  |  |  | 2 | 0 | 0 | 0 | 6 | 4 | 1 | 14 | 25 |
|  |  |  | 3 | 0 | 0 | 3 | 0 | 29 | 9 | 1 | 42 |
| *Onthophagus igualensis* | JF | 1 | 1 | 3 | 0 | 0 | 0 | 0 | 0 | 0 | 3 |
|  |  |  | 2 | 0 | 6 | 5 | 0 | 0 | 0 | 0 | 11 |
|  |  |  | 3 | 5 | 4 | 0 | 0 | 0 | 0 | 0 | 9 |
|  |  | 2 | 1 | 1 | 0 | 0 | 0 | 0 | 0 | 0 | 1 |
|  |  |  | 2 | 8 | 1 | 1 | 0 | 0 | 0 | 0 | 10 |
|  |  |  | 3 | 0 | 3 | 1 | 0 | 0 | 0 | 0 | 4 |
|  | POF | 1 | 1 | 0 | 0 | 0 | 0 | 0 | 0 | 0 | 0 |
|  |  |  | 2 | 0 | 0 | 0 | 0 | 0 | 1 | 0 | 1 |
|  |  |  | 3 | 0 | 0 | 0 | 0 | 0 | 0 | 0 | 0 |
|  |  | 2 | 1 | 0 | 0 | 0 | 0 | 0 | 0 | 0 | 0 |
|  |  |  | 2 | 0 | 0 | 0 | 0 | 0 | 0 | 0 | 0 |
|  |  |  | 3 | 0 | 0 | 0 | 0 | 3 | 0 | 0 | 3 |
| *Onthophagus incensus* | JF | 1 | 1 | 106 | 98 | 86 | 142 | 101 | 127 | 88 | 748 |
|  |  |  | 2 | 102 | 67 | 117 | 206 | 42 | 66 | 114 | 714 |
|  |  |  | 3 | 212 | 63 | 99 | 353 | 138 | 123 | 80 | 1068 |
|  |  | 2 | 1 | 457 | 115 | 237 | 375 | 227 | 107 | 104 | 1622 |
|  |  |  | 2 | 408 | 499 | 347 | 319 | 83 | 103 | 112 | 1871 |
|  |  |  | 3 | 467 | 450 | 565 | 550 | 254 | 123 | 102 | 2511 |
|  | POF | 1 | 1 | 42 | 26 | 26 | 39 | 147 | 33 | 27 | 340 |
|  |  |  | 2 | 45 | 90 | 49 | 52 | 66 | 54 | 85 | 441 |
|  |  |  | 3 | 16 | 17 | 68 | 79 | 75 | 89 | 120 | 464 |
|  |  | 2 | 1 | 39 | 13 | 25 | 86 | 50 | 32 | 54 | 299 |
|  |  |  | 2 | 11 | 36 | 55 | 38 | 76 | 144 | 43 | 403 |
|  |  |  | 3 | 43 | 38 | 64 | 29 | 40 | 29 | 94 | 337 |
| *Onthophagus knulli* | JF | 1 | 1 | 82 | 56 | 58 | 56 | 16 | 22 | 15 | 305 |
|  |  |  | 2 | 65 | 40 | 55 | 138 | 8 | 8 | 15 | 329 |
|  |  |  | 3 | 81 | 19 | 17 | 228 | 46 | 27 | 2 | 420 |
|  |  | 2 | 1 | 520 | 28 | 298 | 368 | 407 | 81 | 38 | 1740 |
|  |  |  | 2 | 417 | 383 | 304 | 462 | 47 | 83 | 28 | 1724 |
|  |  |  | 3 | 402 | 319 | 506 | 340 | 167 | 20 | 78 | 1832 |
|  | POF | 1 | 1 | 2 | 3 | 5 | 6 | 14 | 9 | 8 | 47 |
|  |  |  | 2 | 2 | 5 | 3 | 6 | 18 | 30 | 10 | 74 |
|  |  |  | 3 | 2 | 1 | 11 | 19 | 24 | 46 | 36 | 139 |
|  |  | 2 | 1 | 0 | 0 | 2 | 7 | 9 | 8 | 18 | 44 |
|  |  |  | 2 | 0 | 3 | 3 | 6 | 9 | 13 | 0 | 34 |
|  |  |  | 3 | 1 | 1 | 7 | 18 | 10 | 15 | 28 | 80 |
| *Onthophagus mexicanus* | JF | 1 | 1 | 0 | 0 | 0 | 0 | 0 | 0 | 0 | 0 |
|  |  |  | 2 | 0 | 0 | 0 | 0 | 0 | 0 | 0 | 0 |
|  |  |  | 3 | 0 | 0 | 0 | 0 | 0 | 2 | 2 | 4 |
|  |  | 2 | 1 | 0 | 0 | 0 | 0 | 0 | 0 | 0 | 0 |
|  |  |  | 2 | 0 | 0 | 0 | 3 | 0 | 2 | 0 | 5 |
|  |  |  | 3 | 0 | 1 | 0 | 0 | 0 | 0 | 0 | 1 |
|  | POF | 1 | 1 | 6 | 5 | 32 | 16 | 36 | 242 | 101 | 438 |
|  |  |  | 2 | 6 | 13 | 2 | 36 | 64 | 233 | 546 | 900 |
|  |  |  | 3 | 22 | 1 | 29 | 54 | 150 | 148 | 74 | 478 |
|  |  | 2 | 1 | 5 | 4 | 7 | 11 | 223 | 274 | 111 | 635 |
|  |  |  | 2 | 5 | 5 | 3 | 13 | 133 | 78 | 50 | 287 |
|  |  |  | 3 | 4 | 0 | 6 | 79 | 663 | 689 | 177 | 1618 |
| *Onthophagus sp.* | JF | 1 | 1 | 16 | 0 | 0 | 0 | 0 | 0 | 0 | 16 |
|  |  |  | 2 | 17 | 0 | 0 | 0 | 0 | 0 | 0 | 17 |
|  |  |  | 3 | 0 | 1 | 0 | 37 | 14 | 0 | 0 | 52 |
|  |  | 2 | 1 | 0 | 46 | 0 | 32 | 20 | 12 | 0 | 110 |
|  |  |  | 2 | 0 | 0 | 35 | 20 | 0 | 0 | 3 | 58 |
|  |  |  | 3 | 0 | 0 | 0 | 0 | 15 | 2 | 13 | 30 |
|  | POF | 1 | 1 | 6 | 9 | 0 | 5 | 8 | 2 | 2 | 32 |
|  |  |  | 2 | 6 | 10 | 3 | 0 | 1 | 0 | 0 | 20 |
|  |  |  | 3 | 1 | 1 | 5 | 1 | 0 | 1 | 5 | 14 |
|  |  | 2 | 1 | 12 | 4 | 1 | 12 | 5 | 0 | 1 | 35 |
|  |  |  | 2 | 5 | 11 | 2 | 2 | 1 | 2 | 5 | 28 |
|  |  |  | 3 | 6 | 10 | 8 | 2 | 0 | 0 | 0 | 26 |
| *Phanaeus adonis* | JF | 1 | 1 | 2 | 8 | 11 | 5 | 30 | 36 | 23 | 115 |
|  |  |  | 2 | 3 | 0 | 3 | 12 | 13 | 32 | 36 | 99 |
|  |  |  | 3 | 1 | 3 | 1 | 19 | 10 | 31 | 38 | 103 |
|  |  | 2 | 1 | 12 | 0 | 18 | 20 | 49 | 13 | 17 | 129 |
|  |  |  | 2 | 12 | 15 | 21 | 22 | 23 | 39 | 24 | 156 |
|  |  |  | 3 | 11 | 7 | 11 | 24 | 61 | 52 | 12 | 178 |
|  | POF | 1 | 1 | 0 | 1 | 6 | 7 | 6 | 15 | 19 | 54 |
|  |  |  | 2 | 2 | 1 | 3 | 8 | 10 | 40 | 8 | 72 |
|  |  |  | 3 | 1 | 3 | 41 | 42 | 117 | 18 | 10 | 232 |
|  |  | 2 | 1 | 0 | 2 | 0 | 4 | 14 | 12 | 9 | 41 |
|  |  |  | 2 | 0 | 0 | 2 | 11 | 5 | 3 | 13 | 34 |
|  |  |  | 3 | 1 | 1 | 1 | 7 | 28 | 28 | 7 | 73 |
| *Pseudocanthon clorizans* | JF | 1 | 1 | 0 | 0 | 0 | 0 | 2 | 0 | 0 | 2 |
|  |  |  | 2 | 0 | 0 | 1 | 0 | 0 | 1 | 2 | 4 |
|  |  |  | 3 | 0 | 0 | 0 | 0 | 1 | 9 | 1 | 11 |
|  |  | 2 | 1 | 0 | 0 | 0 | 0 | 4 | 0 | 0 | 4 |
|  |  |  | 2 | 0 | 0 | 0 | 0 | 1 | 1 | 0 | 2 |
|  |  |  | 3 | 0 | 0 | 0 | 1 | 0 | 2 | 0 | 3 |
|  | POF | 1 | 1 | 0 | 0 | 0 | 0 | 0 | 0 | 0 | 0 |
|  |  |  | 2 | 0 | 0 | 0 | 0 | 0 | 0 | 0 | 0 |
|  |  |  | 3 | 0 | 0 | 0 | 0 | 0 | 0 | 0 | 0 |
|  |  | 2 | 1 | 0 | 0 | 0 | 0 | 0 | 0 | 0 | 0 |
|  |  |  | 2 | 0 | 0 | 0 | 0 | 0 | 0 | 0 | 0 |
|  |  |  | 3 | 0 | 0 | 0 | 0 | 0 | 0 | 0 | 0 |
| *Sysiphus mexicanus* | JF | 1 | 1 | 119 | 88 | 78 | 4 | 7 | 2 | 1 | 299 |
|  |  |  | 2 | 83 | 23 | 26 | 43 | 2 | 4 | 1 | 182 |
|  |  |  | 3 | 62 | 35 | 56 | 121 | 19 | 3 | 3 | 299 |
|  |  | 2 | 1 | 299 | 135 | 148 | 108 | 66 | 1 | 10 | 767 |
|  |  |  | 2 | 216 | 200 | 114 | 80 | 8 | 15 | 2 | 635 |
|  |  |  | 3 | 188 | 138 | 156 | 94 | 29 | 5 | 4 | 614 |
|  | POF | 1 | 1 | 0 | 0 | 0 | 0 | 0 | 0 | 0 | 0 |
|  |  |  | 2 | 0 | 0 | 0 | 0 | 0 | 0 | 0 | 0 |
|  |  |  | 3 | 0 | 0 | 0 | 0 | 0 | 0 | 0 | 0 |
|  |  | 2 | 1 | 0 | 0 | 0 | 0 | 0 | 0 | 0 | 0 |
|  |  |  | 2 | 0 | 0 | 0 | 0 | 0 | 0 | 0 | 0 |
|  |  |  | 3 | 0 | 0 | 0 | 0 | 0 | 0 | 0 | 0 |
| Total number of individuals | | | | 5273 | 3696 | 4361 | 5566 | 8167 | 7950 | 6623 | 41636 |
